# Increased angiogenesis is associated with a 32-gene expression signature and 6p21 amplification in aggressive endometrial cancer

**DOI:** 10.18632/oncotarget.3521

**Published:** 2015-03-10

**Authors:** Ingunn M. Stefansson, Maria Raeder, Elisabeth Wik, Monica Mannelqvist, Kanthida Kusonmano, Gøril Knutsvik, Ingfrid Haldorsen, Jone Trovik, Anne M. Øyan, Karl-H. Kalland, Anne Cathrine Staff, Helga B. Salvesen, Lars A. Akslen

**Affiliations:** ^1^ Centre for Cancer Biomarkers CCBIO, Department of Clinical Medicine, Section for Pathology, University of Bergen, Bergen, Norway; ^2^ Department of Pathology, Haukeland University Hospital, Bergen, Norway; ^3^ Department of Obstetrics and Gynaecology, Haukeland University Hospital, Bergen, Norway; ^4^ Department of Radiology, Haukeland University Hospital, Postbox, Bergen, Norway; ^5^ Section for Radiology, University of Bergen, Bergen, Norway; ^6^ Department of Microbiology, Haukeland University Hospital, Bergen, Norway; ^7^ Department of Obstetrics and Gynaecology, Women and Children's Division, Oslo University Hospital, University of Oslo, Norway

**Keywords:** angiogenesis, endometrial cancer, gene expression signature, prognosis

## Abstract

**Background:**

Angiogenesis is a hallmark of cancer. The aim of this study was to explore whether microvessel proliferation is associated with gene expression profiles or copy number alterations in endometrial cancer.

**Methods:**

A prospective series of endometrial carcinomas was studied for angiogenesis markers, gene expression profiles, and gene copy number data. For validation, an independent series of endometrial carcinomas as well as an external cohort of endometrial cancer patients were examined by gene expression microarrays.

**Results:**

Increased microvessel proliferation (MVP) was associated with aggressive tumor features and reduced survival, and a 32-gene expression signature was found to separate tumors with high versus low MVP. An increased 32-gene signature score was confirmed to associate with high-grade tumor features and reduced survival by independent cohorts. Copy number studies revealed that amplification of the 6p21 region was significantly associated with MVP, a high 32-gene score, as well as reduced survival.

**Conclusion:**

Increased MVP was significantly associated with aggressive endometrial cancer and reduced survival. Integrated analyses demonstrated significant associations between increased vascular proliferation, amplification of the 6p21 region, VEGF-A mRNA expression, and the 32-gene angiogenesis signature. Our findings indicate amplification of 6p21 as a possible driver of tumor vascular proliferation in endometrial cancer.

## INTRODUCTION

Although the majority of endometrial cancers are curable by surgery and adjuvant treatment, a subset still displays an aggressive clinico-pathologic phenotype characterized by high histologic grade, lympho-vascular and myometrial invasion, and reduced survival [1]. The mechanisms leading to poor-prognosis subgroups of endometrial cancer, and how to target them, are not known in detail. Recent studies have focused on the importance of genetic alterations in tumor cells, as well as the influence of tumor microenvironment factors [2,3].

We previously reported that several mechanisms appear to be activated and co-exist in aggressive endometrial cancer [4-7], among them tumor angiogenesis [5, 8]. Vascular proliferation, as a novel angiogenesis marker, was associated with features of progressive tumors and impaired prognosis in several cancers [5, 9-11]. Vascular invasion, the presence of tumor cells within vascular channels and a morphologic marker of early metastatic spread, is found more often in tumors with increased angiogenesis and less mature vessels [4,5]. We subsequently showed that active angiogenesis and increased invasive properties by epithelial-mesenchymal transition appeared to co-exist and associate with vascular invasion and reduced survival [12]. These mechanisms might represent an important program for tumor-vascular interactions in aggressive endometrial cancer and might further point to directions for targeted treatment of these tumors. Also, multiple genetic alterations in endometrial carcinoma have been published [13, 14] but little is known about the relationship between genetic changes and activated tumor angiogenesis. Here, we show by integrated analysis of novel angiogenesis tissue markers, gene expression profiling, gene copy number data, and comprehensive clinico-pathologic characterisation that tumor angiogenesis appear to be associated with co-ordinated transcriptional patterns. Thus, increased vascular proliferation was associated with a 32-gene expression signature as well as amplification of the 6p21 region harbouring the VEGF-A gene and a corresponding increase in VEGF-A tissue expression. The 32-gene signature was associated with aggressive tumor features and reduced survival, also by independent validation. Our findings suggest a rationale for anti-angiogenesis treatment in endometrial cancers and that high vascular proliferation might represent a potential biomarker predicting response to VEGF inhibition.

## RESULTS

### Increased angiogenesis is associated with aggressive endometrial cancer

**Figure 1:**
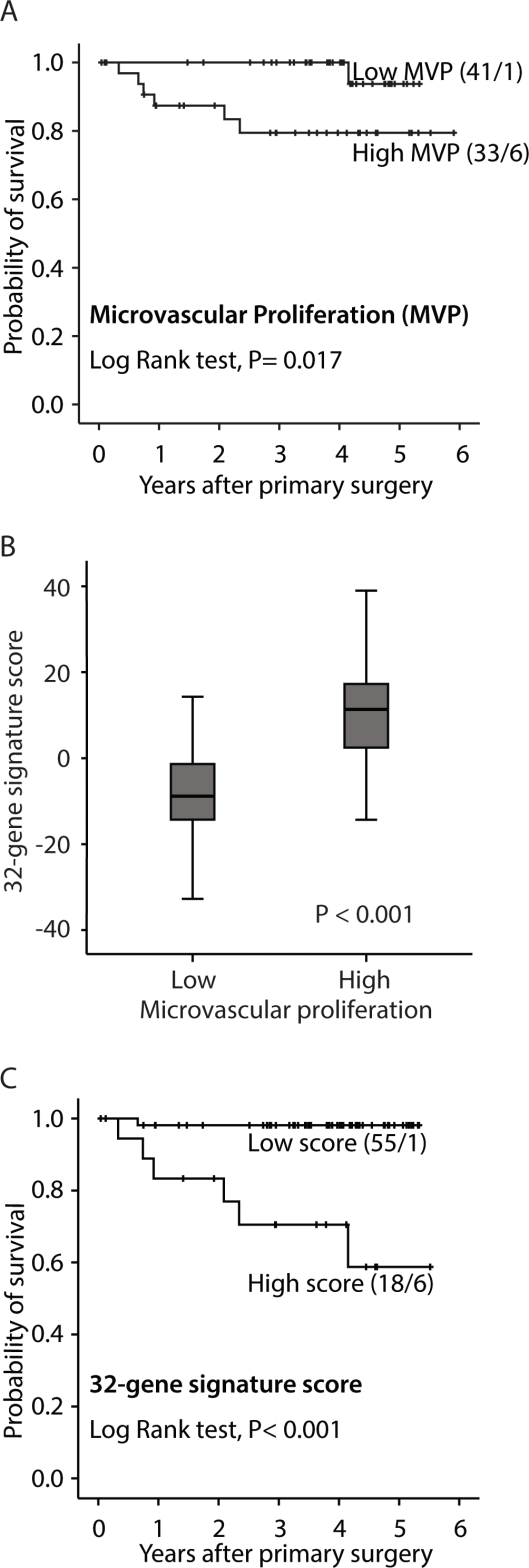
Survival curves by A: high and low microvascular proliferation (MVP), B: high and low vascular proliferation index (VPI) and C: high and low 32-gene signature score. All analyses were done according to the Kaplan-Meier method, (cut-off by median value). Number of cases/number of events in parenthesis.

Median microvessel proliferation (MVP, absolute proliferation) was 2.72/mm^2^ (mean 3.03; range 0-11.25). MVP was significantly associated with an aggressive pathologic phenotype, in particular non-endometrioid histologic type, higher histologic grade, vascular invasion, presence of tumor necrosis, and reduced survival (Table 1; Figure 1A). Correspondingly, median vascular proliferation index (VPI) was 4.5% (mean 5.4%; range 0-18%) in this series. VPI showed similar but slightly weaker associations with histopathologic variables when compared with MVP (Table 1).

**Table 1:** Vascular proliferation (MVP, VPI) in relation to histopathologic features in 77 patients with endometrial carcinoma

Variable	No of patients^a^	Absolute proliferation^b^(MVP)	P-value^c^	Vascular Proliferation Index (VPI)	P-value^c^
Histologic type					
Endometrioid	65	2.4	0.039	4.9	0.043
Non-endometrioid	10	5.1		8.6	
Histologic grade					
Grade 1	16	7.2	0.08	4.1	0.034
Grade 2	41	7.6		5.0	
Grade 3	18	13.2		7.3	
Tumour necrosis					
Absent	34	1.3	0.03	2,8	0.06
Present	41	3.1		6.5	
Mitotic count					
≤10	34	1.7	ns	4,2	0.04
>10	41	3.1		6.4	
Vascular invasion					
0/1 vessel	44	1,4	ns	3.8	0.06
>1 vessel	31	3.4		6.5	
Myometrial infiltration					
≤50	34	2.7	ns	5.3	ns
>50	41	2.7		5.5	
ER					
Positive	54	1.8	ns	3.36	0.022
Negative	19	4.1		6.70	
PR					
Positive	54	1.8	ns	3.66	0.09
Negative	19	4.1		6.53	

a Total number of patients: 77; results available in 75 cases (MVP, VPI) and 73 cases (ER/PR)

b Median value of proliferating vessels pr. mm^2^;

c Mann-Whitney U test

Abbreviations: MVP=microvessel proliferation, VPI= vascular proliferation index, ER=estrogen reseptor, PR= progesterone reseptor

The median microvessel density (MVD) (75 cases) was 50 microvessels/mm^2^ (mean 53; range 0.7-123). Increased MVD was associated with presence of vascular invasion and deep myometrial invasion, but not with any of the other clinico-pathologic variables (data not shown).

Glomeruloid Microvascular Proliferation (GMP), the formation of vascular nests or bodies [15] and assumed to reflect the presence of VEGF-driven angiogenesis [16], was found in 24 tumors (29%), and this feature was associated with presence of tumor necrosis *(P*=0.019) but not with any other clinico-pathologic variables (data not shown).

### A 32-gene expression signature reflecting microvessel proliferation is associated with aggressive tumor features and reduced survival

Genes differentially expressed (cut-off, FDR < 25%) between tumors with high versus low microvessel proliferation (MVP), were included in a vascular proliferation signature of 32 genes (Supplementary Table 1). As expected, the identified 32-gene expression score was associated with MVP (Figure 1B), as well as with features of aggressive endometrial cancer, such as non-endometrioid histologic subtype, high histologic grade, high FIGO stage (Supplementary Figure 1A-C) and reduced survival (Figure 1C).

Interestingly, several of the genes up-regulated in the 32-gene signature (*e.g* TPM1, PDGFB, SERPINH1 and ITGB3) are previously reported to be regulators of vascular proliferation and angiogenesis (Supplementary Table 1); TPM1 is linked to cytoskeleton remodelling critical in angiogenesis [17]; PDGFB is reported to be a potent contributor in angiogenesis by attracting smooth muscle cells as well as VEGF receptors [18]; Colligilin2, the corresponding protein of the gene SERPINH1, has been suggested to take part in neovascularization in gliomas [19]; ITGB3 has been found to take part in endothelial cell adhesion and migration, as part of angiogenesis induced by FOXC2 [20].

Although with a relatively high FDR (<55%) in gene set enrichment analyses (GSEA), gene sets reflecting EGFR/HER2 signaling, TGFβ response [21] and gene ontology categories of angiogenesis stimulation and cell proliferation were significantly enriched in tumors with high microvascular proliferation (MVP), further supporting that our developed vascular proliferation signature is a highly relevant readout for transcriptional alterations regulating angiogenesis. Furthermore, a signature of endothelial cells from invasive endometrial carcinoma [22] and a gene set of HMGA1 targets [23] were enriched in tumors with high MVP. HMGA1 is previously demonstrated to be involved in angiogenesis and cancer progression and is upregulated in response to hypoxia [24].

### The 32-gene signature is associated with other prognostic signatures potentially reflecting angiogenesis

**Figure 2:**
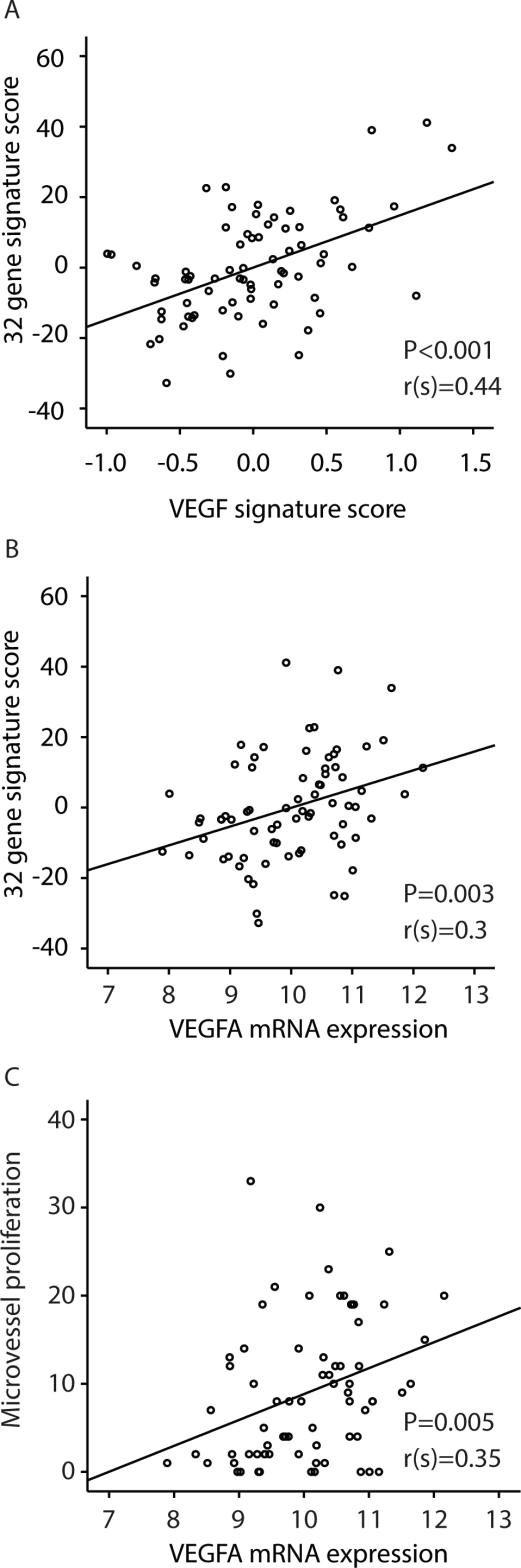
Correlations between A: the 32-gene signature score and a VEGF-signature [25]; B: the 32-gene signature score and VEGF mRNA expression; C: Correlation between microvessel proliferation (MVP) and VEGF mRNA expression. The Spearman rank correlation test was used for bivariate correlations.

We next examined the correlation between our 32-gene score and 6 different gene signatures previously reported to reflect angiogenesis and outcome in a range of cancers: a VEGF signature [25], a vascular invasion signature [26], a wound response signature [27], a hypoxia gene signature [28], a TGFβ gene-response signature of human epithelial cells [29] and a BMI-1 driven stemness related gene signature [30]. All signatures tended to or were significantly correlated to the present 32-gene vascular proliferation signature, as shown in Supplementary Table 2. Figure 2 illustrates this significant correlation between the VEGF-A signature and our 32-gene score (Spearman's rho correlation 0.44; *P<*0.001), as well as the correlation between VEGF-A mRNA and microvessel proliferation in our series (Figure 2A-C).

### Validation of the 32-gene signature score in endometrial cancer

We then explored the 32-gene signature score in an additional independent series of endometrial carcinoma patients (n=37) previously described [31]. There were no differences regarding histologic type, histologic grade, myometrial infiltration or FIGO stage between this series and our primary investigation series (data not shown). In the validation series, the 32-gene score was significantly associated with features of aggressive endometrial cancer, such as non-endometrioid histologic type (*P=*0.009) and high histologic grade (*P=*0.012). A high 32-gene score was significantly associated with progression/recurrence-free survival (Supplementary Figure 2). Also, a high signature score was significantly associated with reduced tumor blood flow (ml/100 ml min^−1^), (both variables categorized by median value), as assessed by functional MRI (82% of tumors with reduced blood flow had high signature score, 18% had low signature score), *P=*0.001).

In the publicly available data set of 111 endometrial carcinomas (NCBI GEO: GSE2109), 23 of the 32 genes in the vascular proliferation signature were found. A high signature score in this data set was significantly associated with non-endometrioid histology, high histologic grade, and high FIGO stage (Supplementary Figure 1D-F), as well as low estrogen and progesterone receptor gene expression (both *P<*0.001).

### Increased 6p21 copy number associates with microvessel proliferation

**Figure 3:**
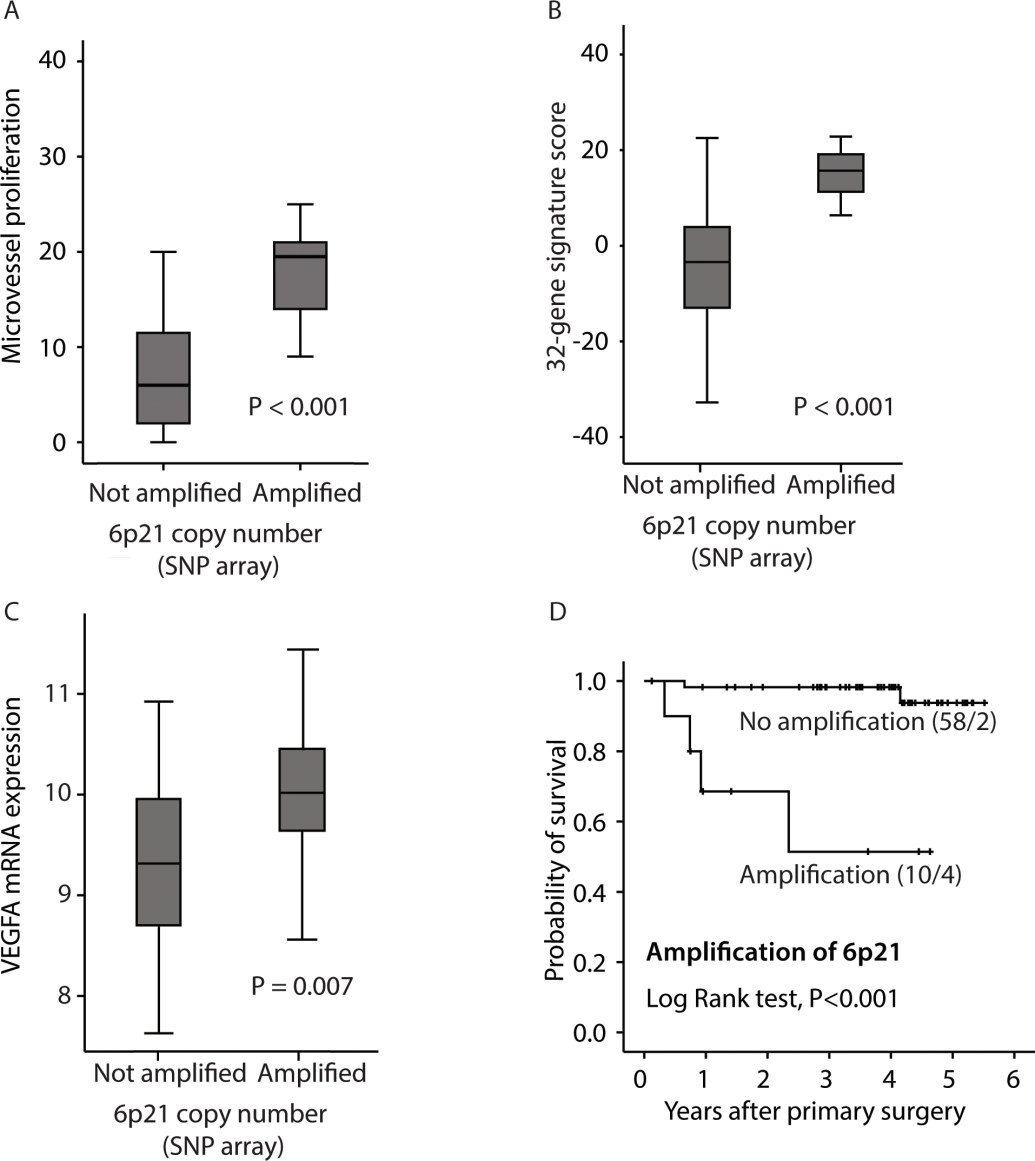
Relation between 6p21 amplification and A: microvessel proliferation (MVP); B: the 32-gene signature score; C: VEGF mRNA expression, and D: disease specific survival according to the Kaplan-Meier method. Number of cases/number of events in parenthesis.

In an analysis of clinico-pathologic factors and significant gene amplifications to predict increased microvessel proliferation, amplification of the 6p21 chromosomal region (present in 10 cases; 15%) was by far most significantly associated with vascular proliferation, both microvessel proliferation (MVP) (Figure 3A) and vascular proliferation index (VPI) (Table 2). Notably, 6p21 amplification was not associated with standard microvessel density (MVD) (*P=*0.41). The 32-gene signature score was significantly elevated in 6p21 amplified cases, and the amplification was associated with reduced patient survival (Figure 3B and D). The 6p21 amplification was significantly associated with more aggressive histopathologic phenotype, such as histologic type, histologic grade, high mitotic count, loss of ER and PR expression, as well as FIGO stage (Table 3).

**Table 2:** Association between 6p21amplification status and measures of vascular proliferation (MPV, VPI)

Variable	No of patients	Microvascular^a^ proliferation (MVP)	P-value^#^	Vascular Proliferation Index (VPI)^*^	P-value^b^
6p21 status				
Amplification −	56	7,56	<0.0001	4,59	<0.0001
Amplification +	10	18,2		11,07	

Abbreviations: MVP=microvessel proliferation, VPI= vascular proliferation index

a Median value of proliferating vessels pr. mm^2^;

b Mann-Whitney U test

**Table 3:** Relationship between 6p21 amplification status and histopathologic features in patients with endometrial carcinoma

Variable	No 6p21 ampl	+6p21 ampl	P-value^c^
Histologic type			
Endometrioid	54 (91)	5 (9)	<0.0001
Non-endometrioid	4 (44)	5 (56)	
Histologic grade			
Grade 1/2	49 (92)	4 (8)	0.002
Grade 3	9 (60)	6 (40)	
Tumour necrosis			
Absent	28 (93)	2 (7)	0.09
Present	30 (79)	8 (21)	
Mitotic count			
≤10	31(100)	0 (0)	0.002
>10	27 (73)	10 (27)	
Vascular invasion			
0/1 vessel	37 (90)	4 (10)	0.15
≥ 2 vessel	21 (78)	6 (22)	
Myometrial infiltration			
< 50	29 (91)	3 (9)	0,24
≥ 50	29 (81)	7(19)	
ER			
Positive	46 (94)	4 (8)	0.009
Negative	12 (67)	6 (33)	
PR			
Positive	48 (94)	3 (6)	<0.0001
Negative	10 (59)	7 (41)	
FIGO stage			
I/II	52 (51)	5 (9)	0.002
III/IV	6 (54)	5 (46)	

c Chi-square test

The VEGF-A gene is located within the amplified 6p21 region and was upregulated in tumors with this amplification (Figure 3C). VEGF gene expression itself (mRNA) was also significantly correlated to microvessel proliferation (Spearman's rho Correlation =0.35, *P=*0.005) (Figure 2C).

### Serum markers of angiogenesis

The serum concentration of several angiogenic factors (VEGF-A, PIGF, GDF-15, sFlt, sEng) were compared to tissue-based angiogenesis markers and clinico-pathologic factors. Myometrial infiltration >50% and high FIGO stage were associated with high levels of GDF-15 in plasma (*P=*0.04 and *P=*0.03, respectively). Amplification of 6p21 was associated with higher GDF-15 concentration in plasma (*P=*0.045, Mann Whitney U -test). Although the sample set is too small to draw definitive negative conclusions, no other clear associations were found, in particular not between these angiogenic factors and tissue based angiogenesis markers.

## DISCUSSION

Tumor-vascular interactions have been increasingly focused as important driver mechanisms in malignant tumors [32, 33]. In particular, angiogenesis is considered a major hallmark and important for the growth and spread of cancers [34, 35], also representing a therapeutic target [36]. Regarding endometrial cancer, we previously reported that active angiogenesis and vascular invasion by tumor cells were related events and important for disease progress in these patients [4, 5]. Here, we confirm in two independent series of endometrial cancer that active angiogenesis, using the novel tissue-based marker microvessel proliferation (MVP), is significantly related to aggressive endometrial cancer.

Gene expression signatures may have a stronger potential to reflect the complexity of cancer biology as compared to the detection of single gene or protein alterations, and thereby be relevant as prognostic and predictive cancer biomarkers. Thus, gene expression signatures are suggested to be of value for improved molecular classification and identification of high-grade subgroups, as published in specific cancer types [37-39], and also recently demonstrated for endometrial carcinomas [7, 26, 40]. Two gene expression signatures (MammaPrint and Oncotype Dx) are approved for clinical utility in breast cancer, predicting risk of recurrence and metastatic disease, and utilized to direct adjuvant therapy to high-risk cases [41].

In our study, a 32-gene angiogenesis signature was found to reflect tumors with high microvessel proliferation, and the corresponding signature score was associated with aggressive tumorfeatures and reduced survival, also validated in two independent data sets. Whereas several genes in the signature are previously associated with vascular biology [19, 20, 42], multiple genes have not been previously linked to angiogenesis. Notably, the genes NFIL3, FSTL3 and FHL3 are up-regulated with highest fold-change in tumors with high vascular proliferation, and these are not directly linked to angiogenesis. NFIL3 is suggested to take part in inhibition of apoptosis [43], potentially also in vasculature, and this mechanism may suggest a link between NFIL3 and angiogenesis. NFIL3 is overexpressed in several cancer types, and is associated with reduced survival in breast cancer [44]. In a mouse model of atherosclerosis, FHL3 was demonstrated to have anti-apoptotic effects in endothelial cells, through up-regulation of Bcl-2 via the PI3K pathway [45]. FSTL3, another gene up-regulated in tumors with high vascular proliferation, is suggested to promote endothelial cell proliferation [46]. Among down-regulated genes in cases with high vascular proliferation, only few of these are previously associated with cancer. The gene *FUK* is reported to be down-regulated in specialized stem cells (side population cells) and associated with chemoresistance in malignant melanoma [47]. The genes FHIT and RBM5 are suggested as tumor suppressor genes [21, 47]. Taken together, mechanisms for how genes in the 32-gene signature might influence angiogenesis in cancer needs to be further explored [21, 47].

The 32-gene score was related to predefined and prognostic gene sets reflecting VEGF activity, wound response, endothelial cell gene expression, epithelial-mesenchymal transition, and a BMI-1 based stemness signature. These findings support a biological relevance of the 32-gene signature, and indicate that tumor angiogenesis is an important feature of aggressive endometrial cancer and should be considered for targeted treatment.

Interestingly, a subset of the cases (15%) showed evidence of 6p21 amplification, which was a strong predictor of increased microvessel proliferation and VEGF-A mRNA expression. VEGF-A is considered an important angiogenic growth factor and is located in the 6p21 chromosomal region [48], although there are some conflicting results about the association between copy number of 6p21 and VEGF expression [49, 50]. In our study, 6p21 amplification was significantly associated with aggressive features of endometrial carcinomas and reduced patient survival. Of note, amplification of 6p21 was also associated with increased serum-concentration of GDF-15, which has been considered an angiogenic factor.

Taken together, our results suggest that increased microvessel proliferation in endometrial cancer is reflected by a 32-gene expression signature which includes several genes possibly related to angiogenesis. Also, 6p21 amplification is a potential driver of angiogenesis in a subset of the tumors, and our findings propose further studies of VEGF amplification as a biomarker for response to anti-angiogenic therapy. This is in line with recent publications on possible targets for treating endometrial carcinoma [2, 51].

## MATERIALs AND METHODS

### Patient series

A primary investigation series (n=77) and an independent validation series (n=37) of endometrial carcinomas (primary tumors) were prospectively enrolled during 2001-2004 and 2009-2011, respectively, as part of a population-based tissue bank. Clinico-pathologic data such as age at time of diagnosis, treatment modality, histologic type and histologic grade, blood vessel invasion, lymphatic vessel invasion, myometrial infiltration, FIGO stage (2009) and disease specific and overall survival were recorded. All samples were reviewed and characterized by one pathologist (I.M.S.) according to published criteria [4]. For the validation series, functional MRI data reflecting tumor blood flow was included, as previously reported [31] Further details on the patient series are given in earlier publications [31, 52].

A publicly available gene expression microarray data set of 111 endometrial carcinomas was obtained from the Expression Project for Oncology (expO:http//expo.intgen.org/geo/home.do. GSE 2109 expO) and applied for validation. GEO accession numbers and clinico-pathologic details on this series are previously described [52].

### Immunohistochemistry

Standard 5 μm sections of formalin-fixed and paraffin-embedded tumor tissues were used. Vascular proliferation was determined as described, using dual staining for Factor-VIII/Ki-67. Briefly, for antigen retrieval, slides were boiled for 20 min in Tris EDTA buffer, PH 9. The slides were incubated in 60 minutes at room temperature with monoclonal antibody Ki-67/MIB-1 (DAKO Norway, Oslo) 1:200, and Factor-VIII polyclonal rabbit antibody (DAKO) 1:800. Ki-67 staining was detected by a biotinylated goat anti mouse secondary antibody 1:100 (E0433, DAKO), and a streptavidin AB-complex (K0391, DAKO) with Fast Blue as chromogen, while Factor-VIII was detected by EV rabbit HRP secondary antibody 1:50, and AEC-peroxidase as a substrate Slides from 75 cases were available and of sufficient quality and quantity for this marker. Staining for ER and PR was performed as previously described [53].

For detection of Glomeruloid Microvascular Proliferations (GMP) [15], slides were treated with proteolytic enzyme (antigen retrieval) in 5 min, followed by incubation with polyclonal Factor-VIII Ab 1:800 in 60 minutes. Staining was done by the rabbit EnVision Kit (DAKO) with diaminobenzidine peroxidase (DAB, DAKO) as substrate. Finally, the sections were counterstained with Dako REAL haematoxylin for 2 minutes. Slides from 76 cases were available for this marker.

### Evaluation of staining results

Microvessel density (MVD) was recorded as previously described [5]. Regarding the novel angiogenesis marker microvascular proliferation (MVP), dividing endothelial cells were recognized by their morphology, their localization, and by distinct Factor-VIII/Ki-67 co-expression. Positive nuclei outside the endothelial cell layer, or within the vessel lumen, were avoided. Vascular proliferation index (VPI) was determined by calculating the ratio between the number of microvessels with proliferating endothelial cells, divided by the total number of Factor-VIII positive microvessels (%), as examined within “hot-spot” areas [5].

Glomeruloid Microvascular Proliferation (GMP) [16] was included because of its prognostic impact found in recent studies of various human cancers [15, 54-56]. Presence of GMP was recorded as described [15] and categorized as being present or absent.

### Oligonucleotide DNA microarray analyses

Fresh tumor tissues were immediately frozen in liquid nitrogen and stored at −80° C. Extracted RNA was hybridized to Agilent Whole Human Genome Microarrays 44k (Cat.no. G4112F), according to manufacturer's instructions (www.agilent.com). Arrays were scanned using the Agilent Microarray Scanner Bundle. Microarray signal intensities were determined using J-Express (www.molmine.no). Median spot signal data were used as intensity measure. The expression data were quantile normalized.

Genes differentially expressed between high and low microvessel proliferation (MVP; by median value) were identified by Significance Analysis of Microarray (SAM) [57]. Gene sets differentially enriched between high and low vascular proliferations were explored by Gene set enrichment analysis (GSEA) [58], based on gene sets available through MSigDB (www.broadinstitute.org/gsea/msigdb).

### Gene expression signatures

Gene expression signature scores were calculated for a 32-gene vascular proliferation signature (see Results) and for a VEGF signature [25], a vascular invasion signature [26], a wound response signature [27], a hypoxia gene signature [28], a TGFβ gene-response signature of human epithelial cells [29] and a BMI-1 driven stemness related gene signature [30]. All expression values were normalized to a common mean and scaled to the same standard deviation before included in the signature score [59]. The 32-gene angiogenesis score was calculated by subtracting the sum of expression values of genes with a low expression from the sum of expression values of genes with high expression in cases with high vascular proliferation. Signature scores were calculated in the same manner for the vascular invasion signature, the hypoxia gene signature, the TGF-β response signature, and the BMI-1 driven gene signature. Mean expression of all genes in the VEGF signature and the wound response signature (the activated genes) constituted the scores for these signatures. For the wound response signature, two out of 208 genes were not found, and three of 153 genes were not found in the TGF-β signature.

### Genomic copy number assessment

Genomic DNA was analyzed for copy number alterations by SNP arrays interrogating 116,204 SNP loci (Affymetrix) and the GISTIC algorithm, as previously described [7, 60].

### Serum markers of angiogenesis

In a subset of 44 endometrial cancer patients, blood samples were analyzed for several angiogenic factors prior to hysterectomy. Details on this patient population are previously published [61]. The following markers were analyzed in serum: VEGF-A, PIGF, GDF-15, sFlt, and sEng [61-63]. Briefly, after 30–60 min at room temperature, serum was obtained by centrifugation and stored at −80°C until assay. Serum concentrations were determined by ELISA (enzyme-linked immunosorbent assay) for human VEGF-A, human PlGF, human sEng and human sFlt1 according to the manufacturer's instructions (R&D systems, Minneapolis, MN). The VEGF and PlGF assays detect free, but not bound, forms of the growth factors. GDF-15 detected in plasma was measured by an immunoradiometric sandwich assay as previously published [61]. The assays were done in duplicate and the results are given in pg/mL serum [62, 63].

### Statistical methods

Comparisons of groups were performed by Pearson's x^2^ test. Mann-Whitney U test was applied for comparison of continuous variables between categories. Spearman correlation coefficient was reported for bivariate correlation between continuous variables. Univariate survival analyses were performed using the product-limit procedure (Kaplan-Meier method), with time of primary surgery as the entry date. Death from endometrial carcinoma or recurrent and progressive disease were applied as end points; death from other causes and no recurrence or progression were censored, respectively. The log-rank (Mantel-Cox) test was used to compare survival curves for different categories of each variable.

## SUPPLEMENTARY MATERIALS, FIGURES AND TABLES


