# Supplementary material for: Increased angiogenesis is associated with a 32-gene expression signature and 6p21 amplification in aggressive endometrial cancer
Source: Oncotarget. 2015 Mar 10;6(12):10634–45. doi: 10.18632/oncotarget.3521 (PMC4496381; doi:10.18632/oncotarget.3521)
Supplement: Supplementary file 1 [file oncotarget-06-10634-s001.pdf]

Increased angiogenesis is associated with a 32-gene expression signature and 6p21 amplification in aggressive endometrial cancer

Supplementary Material

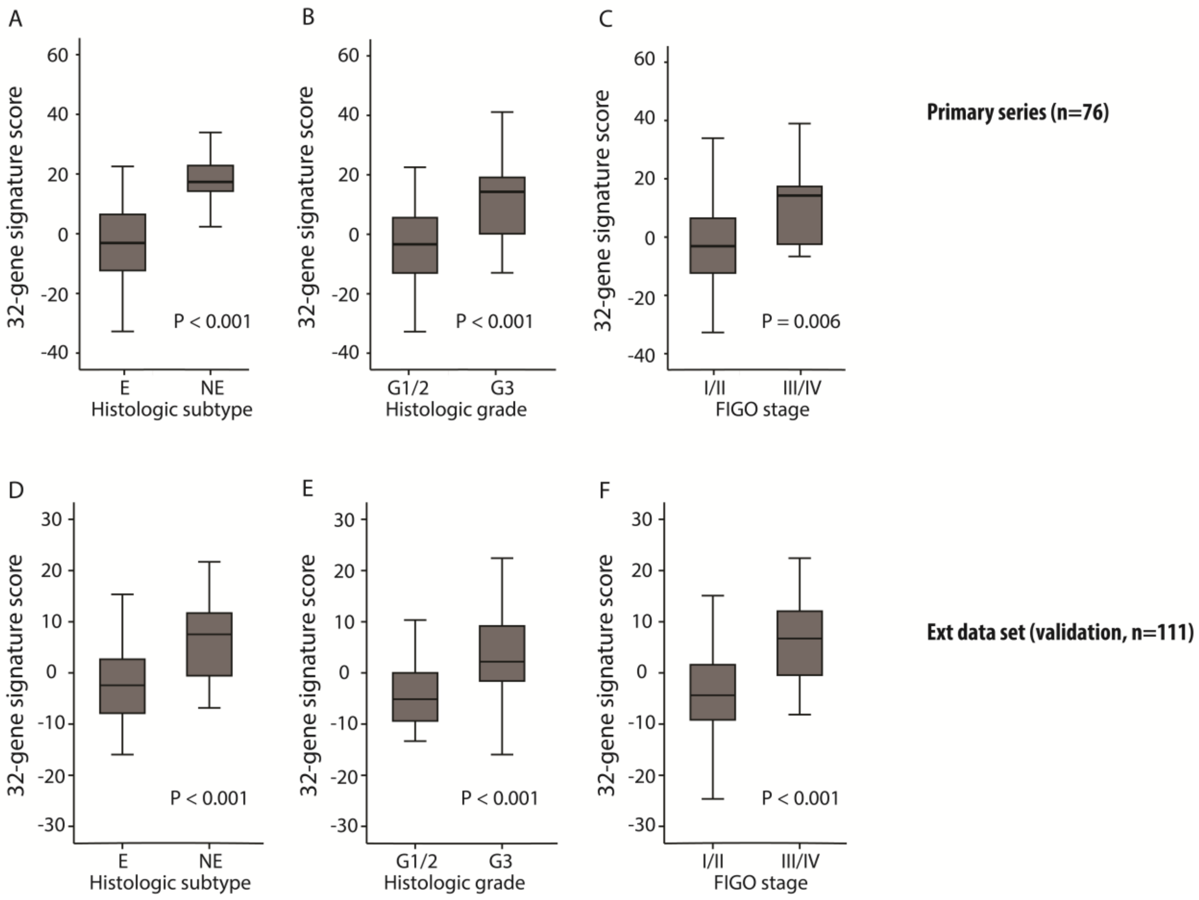

Figure 1: Validation of the 32-gene signature in relation to A: histologic subtype, B: histologic grade, C: FIGO stage in the primary series and correspondingly (D-F) in an external dataset (*NCBI GEO: GSE2109).*

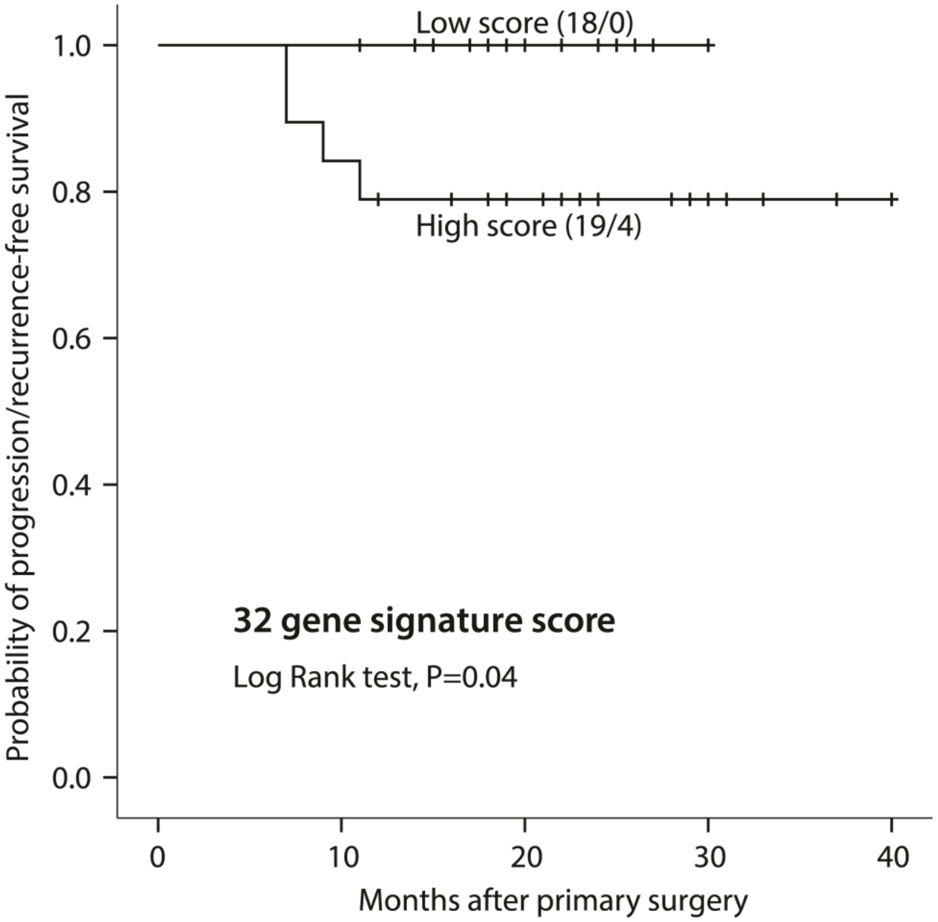

Figure 2: The 32-gene angiogenesis signature and correlation to recurrence-free survival in a validation series according to the Kaplan-Meier method. Number of cases/number of events in parenthesis.

Table S1: Genes differentially expressed between tumors with high versus low vascular proliferation.[a]

Gene name	Systematic name	Description	Fold Change
Upregulated genes			
**NFIL3**	NM_005384	Homo sapiens nuclear factor, interleukin 3 regulated	1.5
**FSTL3**	NM_005860	Homo sapiens follistatin-like 3 (secreted glycoprotein)	1.5
**TPM1**	NM_000366	Homo sapiens tropomyosin 1 (alpha)	1.5
**PDGFB**	NM_002608	Homo sapiens platelet-derived growth factor beta polypeptide (simian sarcoma viral (v-sis) oncogene homolog)	1.4
**FHL3**	NM_004468	Homo sapiens four and a half LIM domains 3	1.4
**SERPINH1**	NM_001235	Homo sapiens serpin peptidase inhibitor, clade H (heat shock protein 47), member 1,	1.4
**ITGB3**	S70348	Homo sapiens integrin beta 3 mRNA	1.3
**ARFRP1**	NM_003224	Homo sapiens ADP-ribosylation factor related protein 1	1.2
**RNASE2**	NM_002934	Homo sapiens ribonuclease, RNase A family, 2 (liver, eosinophil-derived neurotoxin)	1.2
**THC2740750**	THC2740750	Unknown	1.2
**KCNQ3**	NM_004519	Homo sapiens potassium voltage-gated channel, KQT-like subfamily, member 3	1.2
**SERPINB5**	NM_002639	Homo sapiens serpin peptidase inhibitor, clade B (ovalbumin), member 5	1.2
**RNF169**	ENST00000299563	RING finger protein 169. [Source:Uniprot/SWISSPROT;Acc:Q8NCN4]	1.2
**HIST1H2BJ**	BC014312	Homo sapiens histone cluster 1, H2bj, mRNA (cDNA clone MGC:22655 IMAGE:4048288)	1.2
Downregulated genes			
**FLJ21736**	NM_024922	Homo sapiens esterase 31	-1.5
**FHIT**	NM_002012	Homo sapiens fragile histidine triad gene	-1.5
**ZNF75**	NM_007131	Homo sapiens zinc finger protein 75 (D8C6) (ZNF75), mRNA [NM_007131]	-1.5
**PGPEP1**	NM_017712	Homo sapiens pyroglutamyl-peptidase I	-1.4
**C20orf74**	NM_020343	Homo sapiens chromosome 20 open reading frame 74	-1.4
**DDX17**	NM_006386	Homo sapiens DEAD (Asp-Glu-Ala-Asp) box polypeptide 17	-1.3
**ITPK1**	NM_014216	Homo sapiens inositol 1,3,4-triphosphate 5/6 kinase	-1.3
**FUK**	NM_145059	Homo sapiens fucokinase	-1.3
**RBM5**	AF107493	Homo sapiens LUCA-15 protein	-1.3
**LOC442288**	XR_018052	Homo sapiens similar to 60S ribosomal protein L7a	-1.3
**RPL7A**	NM_000972	Homo sapiens ribosomal protein L7a	-1.2
**ERBB2IP**	NM_018695	Homo sapiens erbb2 interacting protein	-1.2
**ABHD17A**	NM_031213	abhydrolase domain containing 17A	-1.2
**NPCDR1**	AF156973	Homo sapiens nasopharyngeal carcinoma down-regulated protein	-1.2
**BC011455**	BC011455	Homo sapiens cDNA clone IMAGE:4177309, partial cds. [BC011455]	-1.2
**SESN3**	NM_144665	sestrin 3	-1.2
**AA609749**	AA609749	TR:G804804 G804804 Hypothetical 4.7 kD Protein; mRNA sequence [AA609749]	-1.2
**AA399656**	AA399656	Ovarian granulosa cell 13.0 kD protein HGR74 (Human); mRNA sequence [AA399656]	-1.1

[a] SAM; significance analysis of microarray; FDR <25%.

**Table S2. Association between the 32-gene angiogenesis signature and other selected gene expression signatures,
related to vascular biology, epithelial-mesenchymal transition and stemness.**

Signature	Spearman's rho Correlation	P-value
VEGF signature [1]	0.44	0.001
Vascular invasion signature [2]	0.19	0.10
Wound response signature [3]	0.33	0.003
Hypoxia gene signature [4]	0.21	0.071
TGFβ gene-response signature [5]	0.55	<0.001
BMI-1 driven signature [6]	0.31	0.008

REFERENCES TO SUPPLEMENTARY TABLE 2

1. Hu Z, Fan C, Livasy C, He X, Oh DS, Ewend MG, Carey LA, Subramanian S, West R, Ikpatt F, Olopade OI, van de Rijn M, Perou CM. A compact VEGF signature associated with distant metastases and poor outcomes. BMC medicine 2009;7:9.
2. Mannelqvist M, Stefansson IM, Bredholt G, Hellem Bo T, Oyan AM, Jonassen I, Kalland KH, Salvesen HB, Akslen LA. Gene expression patterns related to vascular invasion and aggressive features in endometrial cancer. The American journal of pathology 2011;178:861-871.
3. Chang HY, Sneddon JB, Alizadeh AA, Sood R, West RB, Montgomery K, Chi JT, van de Rijn M, Botstein D, Brown PO. Gene expression signature of fibroblast serum response predicts human cancer progression: similarities between tumors and wounds. PLoS biology 2004;2:E7.
4. Chi JT, Wang Z, Nuyten DS, Rodriguez EH, Schaner ME, Salim A, Wang Y, Kristensen GB, Helland A, Borresen-Dale AL, Giaccia A, Longaker MT, Hastie T, et al. Gene expression programs in response to hypoxia: cell type specificity and prognostic significance in human cancers. PLoS medicine 2006;3:e47.
5. Padua D, Zhang XH, Wang Q, Nadal C, Gerald WL, Gomis RR, Massague J. TGFbeta primes breast tumors for lung metastasis seeding through angiopoietin-like 4. Cell 2008;133:66-77.
6. Glinsky GV, Berezovska O, Glinskii AB. Microarray analysis identifies a death-from-cancer signature predicting therapy failure in patients with multiple types of cancer. The Journal of clinical investigation 2005;115:1503-1521.
